# THE ROLE OF FRAILTY IN DEMENTIA EXPRESSION: EVIDENCE FROM CLINICAL-PATHOLOGIC COHORT STUDIES

**DOI:** 10.1093/geroni/igz038.1465

**Published:** 2019-11-08

**Authors:** Lindsay M Wallace, Olga Theou, Melissa K Andrew, David Bennett, Carol Brayne, Kenneth Rockwood

**Affiliations:** 1 Dalhousie University, Halifax, Nova Scotia, Canada; 2 Rush University Medical Centre, Chicago, Illinois, United States; 3 University of cambridge, Cambridge, England, United Kingdom

## Abstract

A growing body of evidence suggests that pathological features of dementia are diverse and don’t wholly explain variability in dementia incidence. Objective: discuss the role of frailty in dementia expression using evidence from clinical-pathologic cohort studies. Methods: Cross-sectional relationships between neuropathology (Braak, CERAD staging), frailty (frailty index at last study visit before death), and dementia diagnosis at death were performed using data from the Rush Memory and Aging Project (MAP) and the Cambridge City Over 75s Cohort study (CC75C). Results: Participants were 89.7±6.2 and 92.2±4.5 years in MAP (n=451) and CC75C (n=177) and mostly female (69.4-70.1%). Most had dementia (52.8%-59.3%). Frailty was normally distributed (mean FI0.42±0.18 in MAP and 0.34±0.16 in CC75C). Frailty was associated with dementia in MAP (OR=1.88, p<0.001) and CC75C (OR=1.30, p=0.03) after controlling for age, sex, time to death, and neuropathology. Longitudinal analysis is in progress. Frailty appears to play a meaningful role in dementia expression.

